# Socio-demographic determinants of the knowledge and attitude of Nepalese healthcare workers toward human monkeypox: a cross-sectional study

**DOI:** 10.3389/fpubh.2023.1161234

**Published:** 2023-05-24

**Authors:** Santa Kumar Das, Abhinav Bhattarai, Simran KC, Sangam Shah, Kiran Paudel, Sakchhyam Timsina, Sunraj Tharu, Laba Rawal, Dawin A. Leon-Figueroa, Alfonso J. Rodriguez-Morales, Joshuan J. Barboza, Ranjit Sah

**Affiliations:** ^1^Tribhuvan University Teaching Hospital, Institute of Medicine, Tribhuvan University, Tribhuvan, Nepal; ^2^Central Department of Public Health, Institute of Medicine, Tribhuvan University, Tribhuvan, Nepal; ^3^Nepal Health Frontiers, Kathmandu, Nepal; ^4^Facultad de Medicina Humana, Universidad San Martín de Porres, Chiclayo, Peru; ^5^Grupo de Investigación Biomedicina, Faculty of Medicine, Fundación Universitaria Autónoma de las Américas - Institución Universitaria Visión de las Américas, Pereira, Risaralda, Colombia; ^6^Master's Program in Clinical Epidemiology and Biostatistics, Universidad Cientifica del Sur, Lima, Peru; ^7^Gilbert and Rose-Marie Chagoury School of Medicine, Lebanese American University, Beirut, Lebanon; ^8^School of Medicine, Universidad Cesar Vallejo, Trujillo, Peru; ^9^Department of Microbiology, Dr. D. Y. Patil Medical College, Hospital and Research Centre, Pune, Maharashtra, India; ^10^Department of Public Health Dentistry, Dr. D.Y. Patil Dental College and Hospital, Pune, Maharashtra, India

**Keywords:** knowledge, healthcare workers, human monkeypox, determinants, attitude

## Abstract

Human monkeypox is an infectious zoonotic disease and since May 2022, there has been a spike in cases worldwide. In this regard, a global health emergency has been declared by the World Health Organization (WHO) on July 23rd, 2022. Although there have been no confirmed human monkeypox cases in Nepal yet, the nation is undeniably at risk of an outbreak. Despite all preventive efforts and preparedness for monkeypox, there still remain several challenges including the literacy and knowledge of our healthcare workers regarding monkeypox. The aim of this study was to assess the level of knowledge and attitude of Nepalese healthcare workers regarding monkeypox. A cross-sectional study was performed on different healthcare workers at Tribhuvan University Teaching Hospital on the month of October 2022 using a set of validated questionnaires used previously in a Saudi Arabian study. An in-person survey was conducted where a total of 220 questionnaires were distributed. The response rate was 93%. Knowledge was categorized into high or low based on the mean knowledge score. The attitude was assessed using a 3-point Likert scale. The association of the knowledge and attitude of the respondents in accordance with their socio-demographics was statistically evaluated using Pearson’s Chi-square test. The mean knowledge score was 13. A larger proportion of the respondents (60.4%) demonstrated a high knowledge and 51.1% demonstrated a positive attitude. Studying monkeypox during medical education possessed a significant difference in the attitude (*p* = 0.025). Knowledge did not vary based on socio-demographic characteristics. Despite almost half a year into the monkeypox outbreak, Nepalese healthcare workers still have an unsatisfying degree of knowledge and a negative attitude regarding its control which shows the need for education and awareness.

## 1. Introduction

Human monkeypox is an epidemic zoonotic infection caused by the Monkeypox virus (MPXV), a double-stranded DNA virus. MPXV belongs to the Poxviridae family, the same family of smallpox, and the clinical signs and symptoms of both align to a greater extent (1). Like general viral illnesses, common symptoms of MPXV are fever, body pain, headache, and fatigue. The disease is particularly identified with rashes on the skin, and generalized lymphadenopathy on the skin, scalp, and genitals (2). Thankfully, life-threatening complications of MPXV, such as pneumonia, secondary skin infections, proctitis, and ocular complications are rare, however, sepsis due to bacterial superinfection has been reported (3). In 2022, a massive spike in human monkeypox cases has occurred that undoubtedly holds possible disastrous consequences if the cases do not subside and instead increase exponentially.

It has been believed that MPXV is a novel virus, but in fact, it is not and has a long history. It was first identified in 1958 at the Statens Serum Institute, Copenhagen, Denmark, in a colony of Asian monkeys *Macaca fasicularis*, kept for the polio vaccine trial (4). The first human outbreak was reported in 1970, in a 9-month-old baby boy in the Democratic Republic of Congo (then called Zaire) where the disease persisted as endemic. Following that, sporadic outbreaks occurred but remained endemic within the African continent (5). The first confirmed human monkeypox case outside endemic Africa was reported in the United States in 2003 via transmission from infected pets imported from Africa. Minute cases were then reported in different countries of the American and Asian continents (6). A larger outbreak occurred for the first time in 2017 in Nigeria with over 70 confirmed cases. The first Asian continent outbreak of human monkeypox reported from Singapore in 2019 was sourced from a Nigerian tourist (7). These cases subsided with only small outbreaks around the globe.

Although in the past, human monkeypox cases have been unnoticeably low, recently the cases are rising internationally creating public health concerns. Since May 2022, an alarming spike in the cases of human monkeypox has been reported from non-endemic countries. Amidst the COVID-19 pandemic, World Health Organization (WHO) has at the same time declared human monkeypox a global health emergency on July 23rd, 2022, considering its outbreak in over 100 nations (8). The first cases in 2022 were reported in the month of May and the transmission rate gradually skyrocketed over the past 6 months. By the end of 2022, human monkeypox cases have been reported from 110 different countries with 83 thousand confirmed cases and the death count has surpassed two hundred. Outbreaks manifested in severe rates in the United States which has reported almost 30 thousand confirmed cases and is the highest among all nations where cases have been confirmed (9). Except for seven countries that have already had a history of sporadic outbreaks, the remaining 103 countries are experiencing the disease for the first time (10). Sadly, the outbreak spread has not been limited only to zoonotic and human-to-human transmission and water-borne transmission have also been detected. In Italy and Thailand, the spike in cases has also been linked to viral dissemination in non-sewer wastewater detected in wastewater surveillance (11, 12).

The current spike in the transmission rates of MPXV throughout the globe has indeed demarcated global public health concerns and the need for continuous effort and implementation of preventive measures. Although cases have been the highest every day, on the mortality side, the death rate seems lower as compared to the previous MPXV strains. The current MPXV strain has manifested a mortality rate of approximately 0.04% which is significantly lower than that of similar viral strains spread over Africa in the past few decades which had a mortality rate of 1–3% (13). However, this fall in the mortality rate can never ease the public health burden of the disease considering the easy person-to-person transmission, spike in transmission rates, and risk to the global economy. At present, there is no definitive treatment available for human monkeypox, however, preventatives using smallpox vaccinations such as Imavanex have shown an efficiency of up to 85% (14, 15). While few countries have not recommended mass vaccinations yet believing that MPXV cases will certainly not explode as did SARS-CoV-2 in the COVID-19 pandemic, few other countries have started purchasing smallpox vaccines to immunize their citizens and prevent MPXV outbreaks (16). Whatever the prediction, the world should indeed be concerned about the possible health and economic consequences considering our past experience during the fatal waves of the COVID-19 pandemic.

In Nepal, MPVX has not yet been reported. Nepal’s southern neighbor India has so far 23 confirmed human monkeypox cases, and considering the open Nepal-India border, Nepal is undeniably at risk of transmission and outbreak (9). Nepal being a developing nation, can possibly suffer from irreversible health and economic crisis as a consequence of the potential pandemic. Essential preparedness and surveillance for monkeypox have already begun in Nepal. With an aim of not missing any monkeypox cases, the National Public Health Laboratory (NPHL), Teku, Kathmandu, Nepal has already established and upgraded its diagnostic tools and laboratory protocols with reference to WHO Standard guidelines on diagnosing monkeypox. A free hotline number has been put forward by the Ministry of Health and Population, Nepal for reporting any febrile symptoms with pox-like lesions, suspicious of monkeypox (17).

Despite all efforts and preparedness, there still remain several challenges including the literacy and knowledge of the citizens on the ongoing health emergency. A report from WHO stated that the lack of sufficient knowledge on monkeypox among the citizens, and specifically healthcare workers is an evident challenge in ensuring public health awareness and safety during the monkeypox outbreak (18). In this regard, several studies have been performed worldwide to assess the knowledge of healthcare workers on monkeypox and it is surprising that most of these studies have reported a low knowledge of health professionals on monkeypox (19–23). So far in Nepal, no studies assessing the knowledge of Nepalese health professionals on monkeypox have been performed. Therefore, this is the first study performed in this context where the Nepalese healthcare workers’ knowledge and attitude toward monkeypox have been studied and its association with their socio-demographics has been established.

## 2. Methods

### 2.1. Study design and ethical approval

A single-centered cross-sectional, in-person survey was carried out at Tribhuvan University Teaching Hospital (TUTH) in Maharajgunj, Kathmandu, Nepal in October 2022. After a thorough review process and revision, ethical approval was obtained from the Institutional Review Board (IRB) of the Institute of Medicine (IOM; Approval number: 184 (6-11) E2).

### 2.2. Study participants and eligibility criteria

All Nepal Medical Council (NMC) and Nepal Health Professionals’ (NHPC) registered health practitioners over 18 years of age and willing to provide consent for participating in the study were included. Those individuals who refused to provide consent were excluded. A convenience sampling strategy was employed to select the study participants. Since authors belonged to varied medical professions within TUTH, the sampling was accomplished by authors themselves who distributed the questionnaire within their respective departments and among colleagues of the same profession. Since our study was aimed to be generalizable for the infinite population, the sample size was calculated as:


$$n=Z^{2}p\;\left(1-p\right)/d^{2}$$

z = standard normal variate = 1.96 at a 95% confidence level.

*p* = expected proportion in population based on previous study or pilot study.

d = desirable error = 5%.

To our knowledge, this is the first study conducted in Nepal. Thus, considering the conservative estimate of 14% with a precision error of 5% and a 95% confidence level. Sample size, *n* = (1.96)^2^ × 0.15 × (1–0.15)/ (0.05) ^2^ = 185.

Taking into account the non-response rate as 10%, the non-response rate = 10% of 185 = 18.5 = 19. Therefore, the minimum sample size = 185 + 19 = 204.

### 2.3. Survey instrument

This survey utilized a structured and validated questionnaire as the survey instrument, previously employed in the study of Alshahrani et al. (24). Although the survey instrument was designed for the study in Saudi Arabia, we performed necessary modifications to match the context of Nepal. The questions were however not translated into the Nepali language. The questionnaire was divided into three sections: (1) socio-demographic details, (2) knowledge questions, and (3) attitude questions. The socio-demographic section comprised seven questions relating to the respondents’ age, gender, profession, years of experience, study, and familiarity with monkeypox during the survey. The knowledge section comprised 22 multiple-choice questions where the respondents had to select either “Yes” or “No.” These knowledge questions were based on the existing facts of monkeypox as per the United States Centers for Disease Control and Prevention (CDC) and prior research performed in this context (25). There were altogether 11 questions assessing the respondents’ attitude toward monkeypox and were supplied with a 3-point Likert scale allowing selections: “Agree,” “Neutral,” and “Disagree.” The attitude questions were based on the respondents’ perspective on the control and prevention of monkeypox cases, the possibility of monkeypox becoming a pandemic, its burden on the healthcare systems, and their interest in learning about novel emerging diseases. A positive response was awarded a point and therefore the attitude scores ranged from 0 (lowest) to 11 (highest). A higher attitude score indicated respondents’ positive attitudes toward the aforementioned questions. To ensure the validity of the modified questionnaire, the survey instrument was pre-tested on 20 healthcare workers. However, these responses were not included in the final responses and were only utilized for enhancing the quality and clarity of the questions.

### 2.4. Data collection process

The data for this survey was aimed to be collected from a wide variety of healthcare workers including doctors, laboratory professionals, epidemiologists, pharmacists, and so on. Representatives from each of the professions were selected and asked to perform an in-person survey among their circle using the questionnaire provided. Prior to the survey, each respondent was asked to provide their consent for participation. An informed consent stating, “I am a Nepali citizen belonging to a medical background and agree to participate in this research.” Was signed. The objectives and expected benefits of the study were clearly explained. Each in-person survey took approximately 5–8 min.

### 2.5. Study variables

The outcome variable in this study was the knowledge of healthcare workers on monkey pox. Altogether 22 knowledge questions were structured and supplied with two selections: “Yes” or “No,” and the accuracy of the answer was marked as a positive point. No negative point was applied. The points that represented the knowledge score were classified as 0 (lowest) to 22 (highest) where higher scores indicated better knowledge. Similarly, each point was assigned a positive attitude response to the attitude question which ranged from 0 to 11.

The socio-demographic characteristics of the respondents that could possibly result in the difference in the knowledge and attitude were assessed. Age was dichotomously ranged as 21–30 years and > 30 years. Gender was classified as either legally male or female. The study variable profession was categorized as: Doctors, and laboratory personnel, epidemiologists, pharmacists, and others (which included optometrists, audiologists, biomedical technicians, and imaging technologists). The years of medical experience were categorized as low or high based on the mean years of experience. Respondents were also dichotomously categorized based on whether they ever studied monkeypox in their academic life. Likewise, the respondents were categorized into two groups: one who had heard about monkey pox before the survey and one who had not heard about monkey pox until the survey. Furthermore, the respondents were divided based on their familiarity with monkey pox as: (1) Never heard, (2) Heard within several days or weeks, and (3) Heard last month or later.

### 2.6. Statistical analysis

For analyzing the data statistically, IBM SPSS Statistics, Version 26 (IBM Corp, Armonk, NY, USA) was used. Categorical study variables were expressed in terms of frequency and percentage. Continuous variables were expressed in terms of mean and standard deviation (SD). Normal distribution was observed for the knowledge scores. Being an exploratory study, the knowledge scores were divided into low and high based on a cut-off. The cut-off was set as the mean knowledge score, i.e., 13. Pearson’s Chi-square test was performed to compare the explanatory and response variables. Similarly, to interrogate the influence of multiple independent variables on knowledge and attitude scores, and to detect the presence of suppressor variables of the univariable analysis, a multivariable analysis was performed. Two models of multivariable analysis were performed, one for attitude and another for knowledge. All variables were subjected to the multivariable analysis with the assignment of one reference category within the variable. The goodness of fit was tested using the Hosmer and Lemeshow test. Result of the multivariable analysis was expressed in terms of adjusted odds ratio and 95% confidence interval. The statistical level of significance was set at a *p*-value of 0.05.

## 3. Results

### 3.1. Socio-demographic characteristics of the respondents

A total of 220 questionnaires were distributed and 217 participants agreed to participate in this survey. 205 complete responses were recorded. A majority of participants who took part in the survey were older than 30 years old (54.1%). There were more male respondents (55.6%) than female respondents (44.4%). Doctors, Laboratory professionals, epidemiologists, and Pharmacists participated in the survey. A majority of the respondents were pharmacists (44.4%) followed by epidemiologists, doctors, and laboratory professionals. The work experience among the healthcare workers differed. A large proportion of the healthcare workers (66.3%) had work experience of less than 7 years. When the respondents were asked if they ever studied monkey pox in their academic life, a majority of them (89.8%) denied. In spite of that, a greater proportion of the respondents (94.1%) had already known about the ongoing monkeypox outbreak at the time of the survey. 5.9% of the respondents had not heard about monkey pox during the survey. Most of the respondents reported that they heard about monkey pox at least a month earlier than the survey (Table 1).

**Table 1:** Socio-demographic characteristics of the respondents.

	Numbers	Percentage
Age in years		
21–30	94	45.9%
31 and above	111	54.1%
Gender		
Male	114	55.6%
Female	91	44.4%
Profession		
Doctors and laboratory personnel	44	21.5
Epidemiologists	44	21.5
Pharmacy	91	44.4
Others	26	12.7
Years of experience		
Low experience	136	66.3
High experience	69	33.7
Studied about monkey pox		
Yes	21	10.2
No	184	89.8
Heard about monkey pox		
Yes	193	94.1
No	12	5.9
First heard		
Haven’t heard	12	5.9
Within several days or week	42	20.5
Last month or later	151	73.7

### 3.2. Relationship between knowledge score and socio-demographic characteristics of the respondents

**Figure 1:**
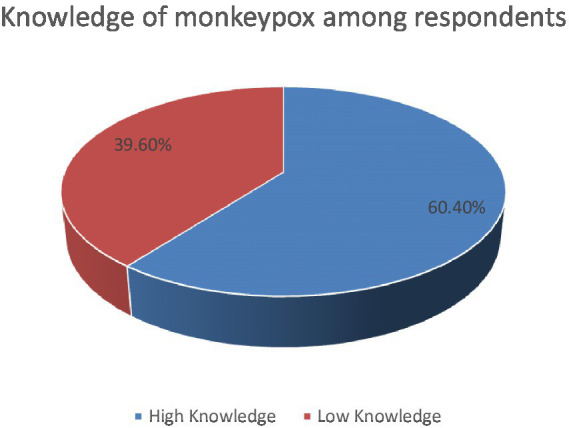
Pie-chart showing the proportion of respondents with high and low knowledge level.

The mean score of respondents’ knowledge on monkeypox was 13 and the levels of knowledge on monkeypox were divided into low and high levels based on that. A majority of the respondents 124 (60.4%) a high knowledge (Figure 1). A higher proportion of respondents (64.9%) over 30 years of age had high knowledge, whereas slightly lesser proportion (55.3%) of respondents below 30 years of age possessed it. This difference in the proportion was however statistically insignificant (*p* = 0.164). Similarly, among those aged 31 years and older, 39 (35.1%) had low knowledge whereas 72 (64.9%) had high knowledge of monkeypox. Although statistically insignificant, a greater proportion of males had higher knowledge as compared to females (61.4% versus 59.3%, *p* = 0.764). Furthermore, the variation of the knowledge was equivalently distributed among different healthcare professions with no significant differences (*p* = 0.856). Overall, the association of age, gender, and healthcare profession with the difference in knowledge of monkeypox among the respondents were statistically insignificant. Interestingly, the knowledge of the respondents who had already heard about monkeypox during the survey wasn’t significantly higher than the knowledge of respondents’ who never heard of it (*p* = 0.095; Table 2). We noticed no significant association of multiple independent variables and the knowledge scores of the respondents (Table 3).

**Table 2:** Relationship between knowledge score and socio-demographic characteristics of the respondents.

Variable	Knowledge level (cut off score = 13)		*p* value
Age in years	Low [*n* (%)]	High [*n* (%)]	
21–30	42(44.7)	52(55.3)	0.164
31 above	39(35.1)	72(64.9)	
Gender			
Male	44(38.6)	70(61.4)	0.764
Female	37(40.7)	54(59.3)	
Profession			
Doctor and laboratory personnel	19(43.2)	25(56.8)	0.856
Epidemiologists	18(40.9)	26(59.1)	
Pharmacists	33(36.3)	58(63.7)	
Others	11(42.3)	15(57.7)	
Years of experience			0.408
Low experience	51(37.5)	85(62.5)	
High experience	30(43.5)	39(56.5)	
Studied about monkey pox			
Yes	10(47.6)	11(52.4)	0.422
No	71(38.6)	113(61.4)	
Heard about monkey pox			
Yes	79(40.9)	114(59.1)	0.095
No	2(16.7)	10(83.3)	
First heard			
Haven’t heard	6(50.0)	6(50.0)	0.741
Within several days or week	16(38.1)	26(61.9)	
Last month or later	59(31.9)	92(60.9)	

**Table 3:** Multivariable analysis for knowledge among respondents.

	*p* value	Adjusted odds ratio	95% C.I. for EXP(B)	
			Lower	Upper
Age 31 and above (Ref. 21–30)	0.072	1.748	0.951	3.214
Profession (Ref. Doctors and laboratory personnel)				
Epidemiologist	0.559	1.314	0.526	3.286
Pharmacy	0.311	1.496	0.686	3.262
Others	0.729	1.198	0.432	3.319
Years of experience (Ref. Low)	0.191	0.652	0.343	1.238
Studied about monkey pox (Ref. Yes)	0.364	1.626	0.569	4.652
Heard Monkey Pox (Ref. Yes)	0.241	4.47	0.365	54.865
First Heard (Ref. Haven’t heard)	0.388			
Within several days or week	0.172	3.064	0.614	15.281
Last month or later	0.257	2.444	0.521	11.456
Gender (Ref. Male)	0.858	1.057	0.578	1.932

### 3.3. Relationship between socio-demographic characteristics and attitude score of the respondents

**Figure 2:**
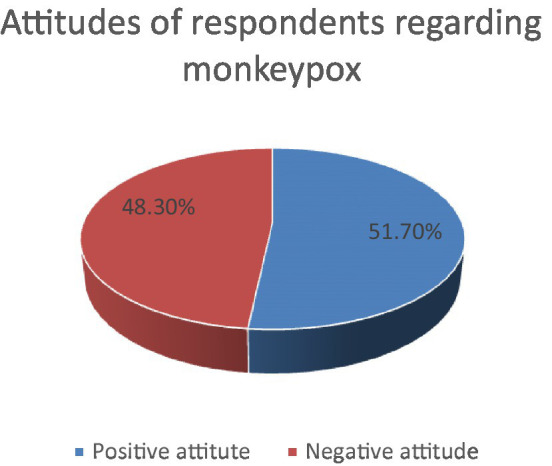
Pie-chart showing the proportion of respondents with positive and negative attitude.

A majority of the respondents (51.7%) had a positive attitude toward monkeypox (Figure 2). When stratified by age group, there were no significant differences between healthcare workers younger and older than 30 years of age (*p* = 0.312). Likewise, both male and female healthcare workers had indifferent attitudes (*p* = 0.988). The attitude further did not differ based on the healthcare profession and years of experience (*p* = 0.197 and *p* = 0.428 respectively). Not surprisingly, those healthcare workers who studied monkeypox in their academic life had a significantly positive attitude toward its control and prevention, and had a greater interest in learning about new emerging diseases (*p* = 0.025). However, the attitude between those who had heard and had not heard about monkeypox until the survey did not differ significantly (*p* = 0.189; Table 4). Our multivariable analysis showed no significant association of the multiple independent variables with attitude and perception of healthcare workers on monkeypox (Table 5).

**Table 4:** Relationship between socio-demographic characteristics and attitude score of the respondents.

Variable	Attitude (cut off score = 13)		*p* value
Age in years	Positive	Negative	0.312
21–30	45(47.9)	49(52.1)	
31 above	61(55.0)	50(45.0)	
Gender			0.988
Male	59(51.8)	55(48.2)	
Female	47(51.6)	44(48.4)	
Profession			
Doctor and lab	18(40.9)	26(59.1)	0.197
Epidemiologists	24(54.5)	20(45.5)	
Pharmacists	53(58.2)	38(41.8)	
Others	11(42.3)	15(57.7)	
Years of experience			0.428
Low experience	73(53.7)	63(46.3)	
High experience	33(47.8)	36(52.2)	
Studied about monkey pox			
Yes	6(28.6)	15(71.4)	0.025
No	100(54.3)	84(45.7)	
Heard about monkey pox			
Yes	102(52.8)	91(47.2)	0.189
No	37(40.7)	54(59.3)	
First heard			
Haven’t heard	3(25.0)	9(75.0)	0.139
Within several days or week	24(57.1)	18(42.9)	
Last month or later	79(52.3)	72(47.7)	

**Table 5:** Multivariable analysis for attitude among respondents.

Variables	*p* value	Adjusted odds ratio	95% C.I. for EXP(B)	
			Lower	Upper
Age 31 and above (Ref. 21–30)	0.194	0.671	0.367	1.225
Profession (Ref. Doctors and laboratory personnel)				
Epidemiologist	0.190	0.543	0.218	1.354
Pharmacy	0.128	0.549	0.254	1.189
Others	0.994	0.996	0.356	2.788
Years of experience (Ref: Low)	0.242	1.465	0.773	2.778
Studied about monkey pox (Ref. Yes)	0.062	0.353	0.118	1.052
Heard monkeypox (Ref. Yes)	0.886	1.110	0.269	4.570
First heard (Ref. Haven’t heard)				
Within several days or week	0.162	0.316	0.063	1.592
Last month or later	0.321	0.454	0.096	2.156
Gender (Ref. Male)	0.699	0.889	0.489	1.615

## 4. Discussion

The findings from our study suggest that although a majority of Nepalese healthcare workers have a higher knowledge regarding monkeypox, they have insufficient critical awareness and perception. Considering null cases of monkeypox in Nepal and no significant public attention yet, it seems obvious that Nepalese healthcare workers are not yet very concerned about the general knowledge and the possible disastrous consequences of monkeypox. However, the general lack of knowledge on monkeypox does not seem to relate to the prevalence of cases in the country as observed in a cross-sectional study performed on Saudi Arabian healthcare workers. Despite frequent cases being confirmed in Saudi Arabia, health practitioners there demonstrated unawareness of monkeypox endemicity, transmission, and its variations from smallpox in an outrageous proportion (24). Similar findings were found in studies conducted in Jordan, Indonesia, Czech Republic, and India where monkeypox has been prevalent (19, 21, 23, 26). In a Nepalese setting, it is therefore questionable whether or not our healthcare workers will develop adequate awareness even when the cases of monkeypox arise and escalate, thus potentially hindering the public health safeguard aims of the nation during the pandemic.

The proportion of Nepalese healthcare workers with high knowledge of monkeypox has however been different from healthcare workers around the world. 64.9% of our survey respondents demonstrated a high knowledge, whereas, using the same survey instrument in a Saudi Arabian study, only 55% of the respondents demonstrated high knowledge (24). Likewise, considerably lower proportions of healthcare workers in Italy (27%), Bangladesh (30.6%), and Indonesia (9%) had high knowledge (19, 20, 27). This difference in the knowledge of monkeypox between Nepalese and international healthcare workers is possibly due to the respective cut-offs and variations in the time of the survey. These studies were performed between March and July 2022, the time when the human monkeypox outbreak had just occurred, whereas, our survey was conducted in the month of October, which is almost 6 months after the outbreak. Despite the long duration of the existence of the monkeypox outbreak and the mass media coverage of the potential pandemic, the knowledge of Nepalese health workers in this context is still unsatisfying.

It is noteworthy that the proportion of Nepalese healthcare workers who studied monkeypox during their education seemed comparably low. Only 10.2% of Nepalese healthcare workers reported that they studied monkeypox during their medical education. A huge proportion (89.8%) of our healthcare workers reported that they did not. In contrast, greater proportions of healthcare workers in Saudi Arabia (18.6%), Italy (42.3%), and Indonesia (17.4%) reported having studied the virus and illness during medical education (19, 20, 24). It implies that either the medical curriculum of Nepal has least emphasized monkeypox, or few proportions of our healthcare workers during their learning period, had been ignorant of the rare viruses and diseases that existed in the past. After all these months of outbreak occurrence and the global health emergency declaration by the WHO, still, 6% of our respondents reported that they had never heard about monkeypox, which indicated that all Nepalese healthcare workers are not keeping themselves up-to-date with the ongoing public health concern. Similarly, 8% of the Indonesian healthcare workers had not heard about monkeypox until the surveyors approached them. At the earliest of the outbreak in May, 56.5% of Saudi Arabian physicians had not ever heard about monkeypox until the survey (24). Likewise, another study revealed that 3.5% of Italian healthcare workers did not know any of the answers on a monkeypox questionnaire and none of the healthcare workers answered all the questions correctly (28). These findings raise a crucial concern regarding the contemporaneity of healthcare workers around the world on monkeypox as well as future disease outbreaks and epidemics.

One of the primary objectives of this study was to relate the socio-demographics of our healthcare workers with their knowledge and attitude regarding monkeypox. None of the socio-demographic characteristics of the respondents defined significant differences in knowledge and our multivariable analysis, showed that none of other variables confounded the association of independent variables with the knowledge and attitude of Nepalese healthcare workers on monkeypox. Both knowledge and attitude did not significantly differ based on age, gender, and experience. However, the attitude did significantly differ between respondents who studied and did not study monkeypox in their medical education. A majority of respondents who had studied monkeypox had a negative attitude, being aware of its potential consequences. These findings reveal that medical education should emphasize diseases of the past that could potentially transform into a pandemic. When using the same survey instrument, the variation in the attitude of our healthcare workers was different from that of Saudi Arabian healthcare workers. In our study, 51.7% of the healthcare workers had a positive attitude toward monkeypox whereas, in the case of Saudi Arabia, it was 15.17% (24). This dissimilarity in attitudes might attribute to the differences in the level of knowledge, comprehension, awareness, and education as discussed earlier. In order to achieve the global aim of monkeypox control or tackle future pandemics, an equivalent knowledge and attitude of healthcare workers throughout the world is a must, and this can certainly be accomplished by the joint collaboration of international medical forums and health ministries in educating their professionals on contemporary outbreaks and global health concerns.

Our research in this context is the first that has been performed in Nepal and holds the ability to aware the health authority of Nepal and South Asia of the contemporaneity of our healthcare workers on the ongoing global health emergency. However, our study holds certain limitations. Foremost, this study was performed in a single center and followed convenience sampling instead of probabilistic, and therefore represents selection and information bias. Although the sample size is sufficient to generate a generalizable interpretation, our sample to an extent might not render the perfect sample of healthcare workers all over Nepal due to our sampling strategy. Additionally, multivariable analyses revealing the association of different variables on knowledge and attitude could not be performed. Since most of the similar studies performed in Jordan, Indonesia, Italy, and Saudi Arabia were conducted at the earliest of the outbreak, we realized the need for up-to-date research on the knowledge and attitude of the healthcare workers in these countries after almost half a year of human monkeypox outbreak that could impart insights on how the knowledge, perception, and comprehension of healthcare workers on monkeypox has changed over time. Lastly, each country including Nepal should develop ideal strategies to enhance the knowledge of their healthcare workers to achieve clinical competency and ability to diagnose and manage human monkeypox cases in the future, and lift off this global health emergency.

## 5. Conclusion

A majority of the Nepalese healthcare workers (60.4%) had a high knowledge of monkeypox and 51.7% had a positive attitude regarding its control. The knowledge did not differ significantly on the basis of socio-demographic characteristics including age, gender, profession, and experience. On the other hand, a significantly lower proportion of healthcare workers who studied monkeypox during their medical education had a positive attitude. Our findings reveal unsatisfying awareness and concern of Nepalese healthcare workers regarding the ongoing global health emergency which needs to be overcome by appropriate education and conferences.

## Data availability statement

The raw data supporting the conclusions of this article will be made available by the authors, without undue reservation.

## Author contributions

SD: concept, design, reviewing, and editing. AB and DL-F: concept, survey instrument development, data collection, manuscript writing, reviewing, and editing. SK: data collection, data entry, manuscript writing, and editing. SS: data collection, reviewing, and editing. KP: data analysis and reviewing. STi: data collection and data entry. STh and LR: data collection. AR-M and JB: data collection, manuscript writing, reviewing, and editing. RS: supervision, reviewing, and editing. All authors contributed to the article and approved the submitted version.

## Conflict of interest

The authors declare that the research was conducted in the absence of any commercial or financial relationships that could be construed as a potential conflict of interest.

## Publisher’s note

All claims expressed in this article are solely those of the authors and do not necessarily represent those of their affiliated organizations, or those of the publisher, the editors and the reviewers. Any product that may be evaluated in this article, or claim that may be made by its manufacturer, is not guaranteed or endorsed by the publisher.
