# Supplementary material for: Socio-demographic determinants of the knowledge and attitude of Nepalese healthcare workers toward human monkeypox: a cross-sectional study
Source: Front Public Health. 2023 May 24;11:1161234. doi: 10.3389/fpubh.2023.1161234 (PMC10244767; doi:10.3389/fpubh.2023.1161234)
Supplement: Supplementary file 1 [file Data_Sheet_1.PDF]

Supplementary material

Questionnaire

Socio-demographic Characteristics of Health Care Workers

1. Age:_____
2. Gender
   - Male ☐
   - Female ☐
3. Profession
   - Doctor ☐
   - Nurse ☐
   - Laboratory personnel ☐
   - Radio technologist ☐
   - Epidemiologist ☐
   - Pharmacist ☐
   - Optometrist ☐
   - Others: please specify……………..
4. Medical practice experience in years………..
5. Information about human monkey pox during medical education
   - Yes ☐
   - No ☐
6. Heard about human monkey pox before
   - Yes ☐
   - No ☐
7. When was the first time you heard information about monkey pox
   - I did not hear about it ☐
   - Within several days or weeks ago ☐
   - Within the last month or later ☐
   -
   - **Knowledge Questions**

1. Is monkey pox prevalent in Western countries?
   - Yes
   - No
2. Is monkey pox prevalent in South East Asian countries?
   - Yes
   - No
3. There are many human monkey pox cases in Nepal?
   - Yes
   - No
4. Is monkey pox a viral disease infection?
   - Yes

- No

5. Is monkey pox a bacterial disease infection?
- Yes
- No

6. Is monkey pox easily transmitted human-to-human?
- Yes
- No

7. Could monkey pox be transmitted through a bite of an infected monkey?
- Yes
- No

8. Travelers’ from America and Europe are the primary source of imported cases of monkey pox?
- Yes
- No

9. Do monkey pox and smallpox have similar signs and symptoms?
- Yes
- No

10. Do monkey pox and chickenpox have similar signs and symptoms?
- Yes
- No

11. A flu-like syndrome is one of the early signs or symptoms of human monkey pox?
- Yes
- No

12. Rashes on the skin are one of the signs or symptoms of human monkey pox?
- Yes
- No

13. Papules on the skin are one of the signs or symptoms of human monkey pox?
- Yes
- No

14. Vesicles on the skin are one of the signs or symptoms of human monkey pox?
- Yes
- No

15. Pustules on the skin are one of the signs or symptoms of human monkey pox?
- Yes
- No

16. Is diarrhea one of the signs or symptoms of human monkey pox?
- Yes
- No

17. Lymphadenopathy (swollen lymph nodes) is one clinical sign or symptom that could be used to differentiate between monkey pox and smallpox cases?
- Yes
- No

18. One management option for symptomatic monkey pox patients is to use paracetamol?
- Yes
- No
19. Are antivirals required in the management of human monkey pox patients?
- Yes
- No
20. Are antibiotics required in the management of human monkey pox patients?
- Yes
- No
21. People who got the chickenpox vaccine are immunized against monkey pox?
- Yes
- No
22. There is a specific vaccine for monkey pox?
- Yes
- No
23. There is a specific treatment for monkey pox?
- Yes
- No

Attitude questions

1. I am confident that the world's population can control monkey pox worldwide.
- Strongly agree, Agree, Strongly disagree, Disagree, Neutral

2. I am confident that in Nepal the local population can control monkey pox locally.
- Strongly agree, Agree, Strongly disagree, Disagree, Neutral

3. I think that there are currently enough prevention and control measures for monkey pox.
- Strongly agree, Agree, Strongly disagree, Disagree, Neutral

4. I have bad feelings toward the monkey pox virus that it might become a worldwide pandemic.
- Strongly agree, Agree, Strongly disagree, Disagree, Neutral

5. I think that monkey pox can add a new burden on the healthcare system of the affected countries.
- Strongly agree, Agree, Strongly disagree, Disagree, Neutral

6. I think monkey pox can be transmitted in Nepal.
- Strongly agree, Agree, Strongly disagree, Disagree, Neutral

7. I think that mass media coverage of monkey pox may influence its worldwide prevention.
- Strongly agree, Agree, Strongly disagree, Disagree, Neutral

8. I am interested in learning more about monkey pox.
- Strongly agree, Agree, Strongly disagree, Disagree, Neutral

9. I am interested to learn more about the epidemiology of the new emerging diseases.
- Strongly agree, Agree, Strongly disagree, Disagree, Neutral

10. I am interested in learning more about Travel Medicine.
- Strongly agree, Agree, Strongly disagree, Disagree, Neutral

11. I think that it is dangerous to travel to countries with monkey pox epidemic.
- Strongly agree, Agree, Strongly disagree, Disagree, Neutral
